# Effect of Surface Microstructure on the Heat Dissipation Performance of Heat Sinks Used in Electronic Devices

**DOI:** 10.3390/mi12030265

**Published:** 2021-03-04

**Authors:** Yuxin You, Beibei Zhang, Sulian Tao, Zihui Liang, Biao Tang, Rui Zhou, Dong Yuan

**Affiliations:** 1 Guangdong Provincial Key Laboratory of Optical Information Materials and Technology & Institute of Electronic Paper Displays, South China Academy of Advanced Optoelectronics, South China Normal University, Guangzhou 510006, China; 2018023198@m.scnu.edu.cn (Y.Y.); 2016010185@m.scnu.edu.cn (B.Z.); tangbiao@scnu.edu.cn (B.T.); zhourui@m.scnu.edu.cn (R.Z.); 2 SCNU-TUE Joint Lab of Device Integrated Responsive Materials (DIRM), National Center for International Research on Green Optoelectronics, South China Normal University, No 378, West Waihuan Road, Guan zhou Higher Education Mega Center, Guangzhou 510006, China; 3 Department of Mechanical Engineering, Guangdong Technical College of Water Resources and Electric Engineering, Guangzhou 510925, China; 4 Kone Elevators (China) Co. Ltd., Kunshan 215347, China; zihui.liang@kone.com

**Keywords:** heat sinks, surface microstructures, heat dissipation performance, thermal radiation, surface roughness

## Abstract

Heat sinks are widely used in electronic devices with high heat flux. The design and build of microstructures on heat sinks has shown effectiveness in improving heat dissipation efficiency. In this paper, four kinds of treatment methods were used to make different microstructures on heat sink surfaces, and thermal radiation coating also applied onto the heat sink surfaces to improve thermal radiation. The surface roughness, thermal emissivity and heat dissipation performance with and without thermal radiation coating of the heat sinks were studied. The result shows that with an increase of surface roughness, the thermal emissivity can increase up to 2.5 times. With thermal radiation coating on a surface with microstructures, the heat dissipation was further improved because the heat conduction at the coating and heat sink interface was enhanced. Therefore, surface treatment can improve the heat dissipation performance of the heat sink significantly by enhancing the thermal convection, radiation and conduction.

## 1. Introduction

With the increasing of the integration density of electronic devices, the high heat flux generated inside the devices has become a critical threaten to the reliability of performance [1]. Therefore, the question of how to achieve effective heat dissipation is always a hot research topic [2].

Finned heat sinks, dissipating heat by natural convection or forced convection with fans [3], are widely used in cooling electronic devices. In these cooling systems, air is used as a coolant because it is easy to obtain and due to the simplicity, high reliability and low cost of the required equipment [4]. As the demand for cooling intensity increases, many heat sinks with diverse fin structures were reported, including flat-plate fin [5,6], pin fin [7,8], interrupted fin [9], slotted fins [10], etc. Many attempts in optimizing the structure on heat sinks, for instance, the width, height, distribution and shape of fins [3,5,8,11,12] have been made. Another way is to change the material of the heat sink, including the fins and substrate [13,14]. Porous medias such as micro tubes [15,16], sintered metal [17,18] and metal foam [19,20] are very popular fin materials in research because they have very large convection area. The orientation direction of the heat sink in application [21,22] and airflow condition [23,24] were also found to have impact on cooling efficiency.

With the development of micromachining technology, various microstructures, including ribs [25,26], dimpled surfaces [27,28,29], surface with arrays of protrusions [30] and roughened surfaces [7,31] have been applied on heat sinks and have shown promising results in enhancing heat dissipation. These microstructures can enhance the convective heat transfer significantly without substantial increasing the pressure drop of the airflow. The literature mainly focus on the heat convection enhancement due to the surface area increase and turbulence flow of the coolant caused by the microstructures, while the contribution of the thermal radiation which plays an important role [32] is always ignored. On the other hand, the fabrication of these surface microstructures normally relies on processes that are quite costly and ineffective, such as welding, embossing [30], milling [33], laser sintering [31] and so on.

In this work, we propose using common and simple surface treatment methods, including chemical polishing, chemical coarsening, mechanical shot peening and chemical oxidation, to fabricate surface microstructures with controllable roughness on flat-plate fin heat sinks. The influence of microstructures and roughness on the thermal emissivity and thermal convection of the heat sinks were studied experimentally. A thermal radiation coating was also implemented, and the interaction between the roughened surfaces and coating were also investigated.

## 2. Materials and Methods

### 2.1. Heat Sink Sample

**Figure 1:**
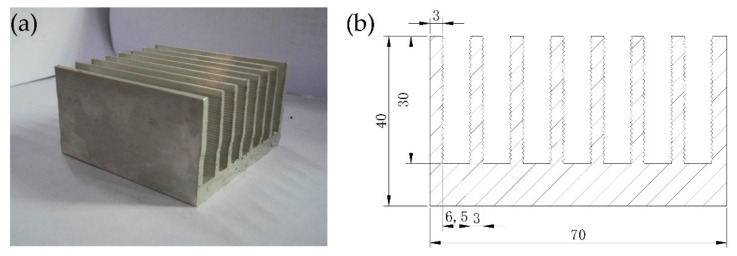
Heat sink sample used for surface treatment: (**a**) photo of the untreated heat sink; (**b**) structural parameters of the heat sink (unit: mm).

The heat sink samples used in this study were made of 6063 aluminum alloy through the extrusion process, as shown in Figure 1a. The structural parameters of the heat sinks are shown in Figure 1b, the length of each sample was 70 mm. In addition, the fin surfaces had a corrugated structure, which can enhance the heat dissipation. All the heat sink samples were cleaned to remove the oil and oxide layer. The samples were first washed by an alkaline solution with 50 g/L Na_2_CO_3_, 15 g/L Na_2_CrO_4_ and 2.5 g/L NaOH at 100 °C for 4 min. Then the transformed aluminum oxide layer and the residual alkaline solution were washed away by immersion into a nitric acid solution (250 mL/L) for 3–4 s. After being washed by water, a clean and uniform surface was obtained that was ready for surface treatments.

### 2.2. Surface Treatment Methods

The heat sinks were treated by chemical and mechanical methods to get different surface microstructures. The methods including chemical polishing, chemical coarsening, chemical oxidation, mechanical shot peening and thermal radiation coating.

The chemical polishing used a strong acid polishing fluid 031003 purchased from Weihai Runking chemical development institute, Weihai, China. The samples were immersed in the polishing fluid with shaking at 100 °C for 90 s.

The chemical coarsening used a solution with 310 g/L NaOH, 45 g/L Na_3_PO_4_ and 20 g/L NaF, the samples were immersed into the solution at 70 °C for 2 min. Then washed away the black aluminum oxide layer by immersing into a weak acid solution until the surfaces became clean.

The chemical oxidation used an alkaline solution with 50 g/L Na_2_CO_3_ and 15 g/L Na_2_CrO_4_, the samples were immersed into the solution at 100 °C for 8 min. Then washed away the residual oxide by immersing into a weak acid solution until the surfaces became clean. After chemical treatments, all the samples were washed with water and then dried.

For the mechanical shot peening, a manual air peener was used. The shot with a diameter of 0.6 mm was made of 410 stainless steel, ejection speed was about 60 m/s, and coverage was 200%.

The thermal radiation coating was a radiant cooling paint purchased from Beijing Ronglihengye Technology Co. Ltd., Beijing, China. The paint was sprayed onto the heat sink surfaces by a spray gun linked with compressed air. The thickness of the coating was controlled by the number of layers. For each layer, the thickness was about 0.8 μm. During spraying, apply the next layer after the previous layer was completely dry until the desired coating thickness was reached.

### 2.3. Characterization and Measurements

#### 2.3.1. Heat Sink Surface Roughness and Emissivity Test

The surface roughness of the samples was measured by a TIME^®^ TR200 hand-held roughness meter (Beijing Time High Technology Co. Ltd., Beijing, China). In the experiment, nine evenly distributed points were measured on the heat sink surfaces, and the mean value and uncertainty were calculated.

**Figure 2:**
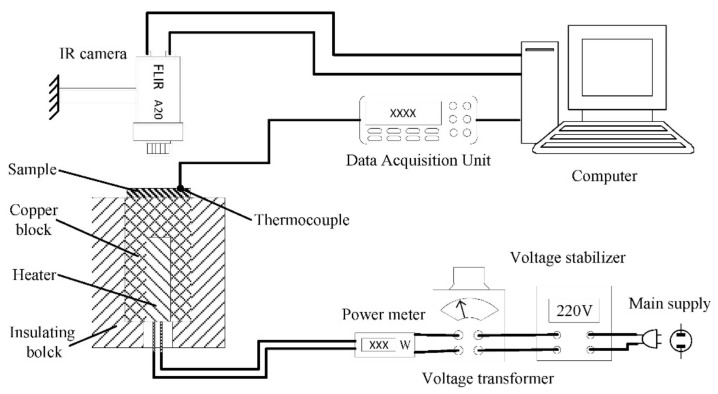
Heat sink surface thermal emissivity test system with infrared (IR) camera.

The thermal emissivity of the heat sink surfaces was tested by an IR camera (FLIR-A20, FLIR Systems Inc., Portland, OR, USA), the experimental setup is shown in Figure 2. The IR camera determines the surface temperature of a sample according to the infrared radiation received from the measured surface. Even at the same temperature, the measured temperature will be different due to the difference in the thermal emissivity of the sample surface. In this experiment, a copper block with an embedded cartridge heater was used as heat source. The temperature measured by a thermocouple fastened onto the sample surface was used as the reference value. The setting of thermal emissivity value was changed in the IR camera until the measured temperature was the same as the temperature measured by the thermocouple, and this value was the thermal emissivity of the sample surface. The size of the samples cut from the heat sink fins was 20 mm × 20 mm × 3 mm. The measuring distance was 0.3 m and the ambient temperature was 26.5 °C. In order to verify the accuracy of thermal emissivity measured by this method, the glass surface and silver film with a known thermal emissivity were tested, and the measured thermal emissivity of these two surfaces were 0.94 and 0.04 respectively, which were consistent with the actual value. Therefore, it was feasible to test the thermal emissivity by this method.

#### 2.3.2. Heat Dissipation Performance Test

**Figure 3:**
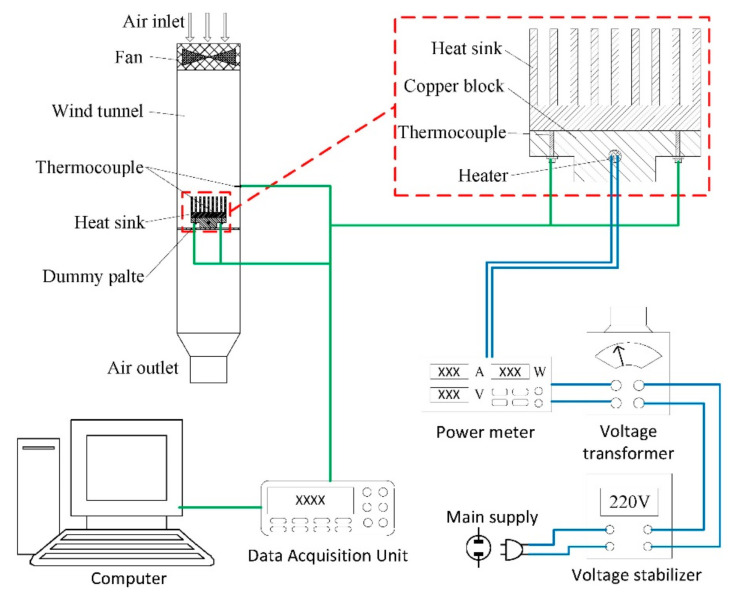
Heat dissipation performance test system with wind tunnel.

In order to study the effect of surface roughness on heat dissipation performance under forced convection, a wind tunnel experiment with a turbulent airflow was carried out. The experimental setup is shown in Figure 3. Since a very good forced convection will hide the effect of radiation heat dissipation, the heat sinks were set vertical to the air velocity vector to compromise the heat convection and radiation. Thus, the influence of surface microstructure on thermal radiation can be clearly presented. The wind tunnel was made of aluminum alloy, width and length was 120 mm and the height was 500 mm. the heat sink samples were place on an air-permeable plate which was 300 mm away from the air inlet. A fan was used to generate airflow in wind tunnel. The power of the fan was 36 W and the wind speed was 15 m/s.

Joule heating was provided to simulate the heat source through a copper block with an embedded cartridge heater capable of up to 100 W (the input power was controlled by a voltage transformer). The heating surface was pressed against the heat sink’s bottom surface by screws to ensure the effective contact. The heating power used in this study was 10, 30, 50, 70 and 90 W.

Four K-type thermocouples were attached on the bottom surfaces of the heat sinks to measure the temperature of them. The four thermocouples were distributed in the four corners of the heat sinks with 10 mm distance to the nearest two edges, and distance between two neighboring thermocouples was 50 mm, as shown in the zoom-in part of Figure 3. The average value of temperatures measured by these four thermocouples was identified as the heat sink temperature. Another thermocouple was placed in the wind tunnel to measure the air temperature, its position was just above the heat sinks. An Agilent data acquisition unit 34,970 A (Agilent Technologies Inc., California, CA, USA) was used to collect and transmit all the thermal data of the thermocouples.

## 3. Results and Discussion

### 3.1. Surface Properities of Heat Sinks with Different Treatments

#### 3.1.1. Surface Roughness

**Figure 4:**
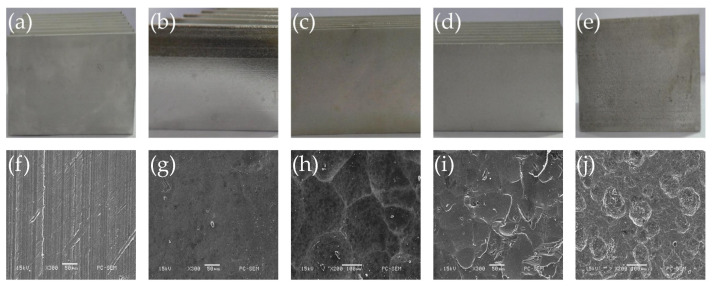
Surfaces of heat sinks with different treatment: photos of heat sinks (**a**) without treatment, (**b**) chemical polishing, (**c**) chemical coarsening, (**d**) mechanical shot peening and (**e**) chemical oxidation; SEM images of heat sinks surfaces (**f**) without treatment, (**g**) with chemical polishing, (**h**) chemical coarsening, (**i**) mechanical shot peening and (**j**) chemical oxidation.

The images of heat sinks after different treatments are shown in Figure 4 and the measured surface roughness is listed in Table 1. As shown in Figure 4a,f, the heat sink surface without treatment is quite smooth. From the SEM image, we can see some parallel scratches along the length direction. These were caused by the die during the extrusion process. The roughness *R*a of untreated surface is 1.24 μm, which is the normal value for extruded aluminum alloy. After chemical polishing, the heat sink surface becomes more smooth (Figure 4g), the roughness reduced to 0.27 μm. This is because the extrusion scratches with higher height were etched by the strong acid, and the surface oxidation layer and crystal layer also gradually dissolved, exposing the original surface of the aluminum alloy. After chemical coarsening, the surface of the sample shows a frosting appearance, the original extrusion scratches on the surface disappeared and the isotropic pitting structure appeared (Figure 4h). The structure is uniform and prominent on the surface, the roughness increased to 4.6 μm. After mechanical shot peening, the original surface was deformed obviously, and the scratches also disappeared. As shown in Figure 4i, the microstructure is similar to the crushing marks, which showed irregular microscopic defects, and the surface roughness increased to 5.63 μm. Figure 4j presents a quite rough surface after chemical oxidation, which is covered by dense corrosion pits. The size and depth of the pits are not uniform and part of them are overlap with each other. This is because the original surface with scratches were not uniform, which lead to the difference in reaction speeds at different places, where the roughness increased to 16.5 μm.

#### 3.1.2. Thermal Emissivity

**Figure 5:**
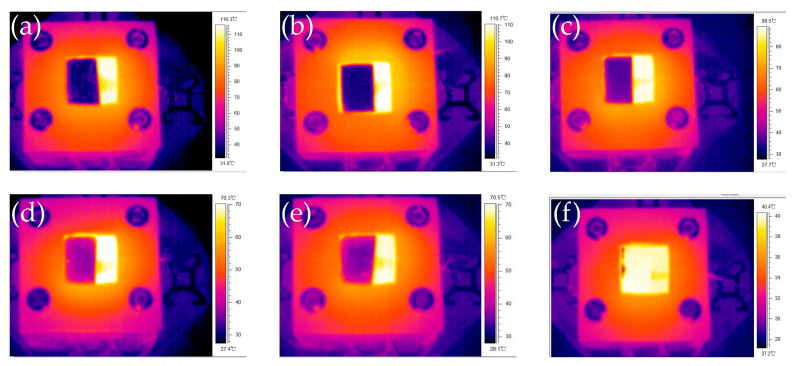
The IR thermal images of heat sink surfaces with different treatments at 40 °C: (**a**) without surface treatment; (**b**) chemical polishing; (**c**) chemical coarsening; (**d**) mechanical shot peening; (**e**) chemical oxidation; (**f**) thermal radiation coating.

The thermal emissivity of the surface is not only related to material composition and structure, but also related to the surface properties. In this study, we found the thermal emissivity by changing its value in the IR camera setting until it fit the tested temperature. The IR thermal images of heat sinks with different treatments are shown in Figure 5. The square part in the center of the images is the surface of the sample. It contents a dark part and a bright part. The bright part is the heat-conducting tape, which was used to fasten the thermocouple. Its emissivity is much higher than the sample surfaces, so it shows a higher temperature than real since the thermal emissivity value setting in the IR camera was lower. The dark part is the sample surface, the surface temperature of all samples is 40 °C in this test, but the color is quite different with each other. The chemical polished surface which has the lowest surface roughness has the darkest color, this indicated that the thermal emissivity difference between it and the heat-conducting tape is the largest, so it has the lowest thermal emissivity. With the increase of surface roughness, the sample surface becomes brighter gradually, so the thermal emissivity also increase.

The thermal emissivity of heat sinks with different surface treatments at 40 °C is listed in Table 2. Except for the thermal radiation coating, other surfaces have the same material, but the surface morphology and roughness are different, so the roughness of surface has great influence on its thermal emissivity. When the surface roughness increase, the thermal emissivity also increase. When the surface roughness is low, the increase in thermal emissivity is not so significant, For example, the surface roughness of the sample without treatment is about 5 times higher than the chemically polished sample, but the thermal emissivity only increased from 0.1 to 0.11. When the surface roughness is high, the increase in thermal emissivity is much more significant, the thermal emissivity of chemical oxidation surface is about 2.5 times of the untreated surface.

The increase of thermal emissivity with larger surface roughness is because of the decrease of light reflection. According to Kirchhoff’s law (emissivity), at a given temperature, for a given wavelength, the ratio of specific emissivity to absorption of all objects is the same, and is equal to the specific emissivity of the ideal black body at that temperature and wavelength. In other words, the higher the absorption rate of the material surface, the higher the thermal emissivity. For a smooth and flat surface with very low roughness, incident light touches the surface only once, some of the light is absorbed and the rest is reflected into the atmosphere directly. At this time, the surface has the highest reflectance and lowest absorbance, so it also has the lowest thermal emissivity. However, when the surface has rough structure, the incident light is reflected on the surface diffusely, and the reflected light may touch the rest of the surface again, so the absorption may happen more than once. The opportunity of IR light absorption of the surface is increased, the absorption rate is increased, and the emissivity is also increased. Therefore, the increase of surface roughness is beneficial to improve the surface thermal emissivity.

After the thermal radiation coating was applied, the surface material of the samples were changed to a thermal material with near-blackbody emissivity, so the thermal emissivity reached 0.98. In the IR image shows in Figure 5f, the color of the sample with coating is even brighter than the heat-conducting tape, because the coating has higher emissivity.

### 3.2. Heat Dissipation Performance of Heat Sinks with Different Surface Treatments

**Figure 6:**
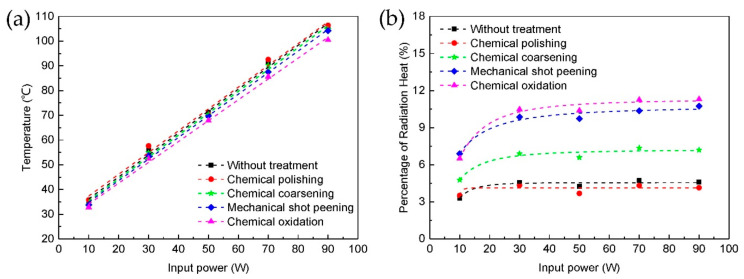
Performance of heat sinks with different surface treatment in wind tunnel under different heating power: (**a**) temperature of heat sinks, (**b**) percentage of radiation heat.

The heat dissipation performances of the heat sinks with different surface treatments were tested in a wind tunnel. The temperatures of heat sinks under different heating power are shown in Figure 6a, all the temperatures were measured after the whole system reached thermal equilibrium state. It can be seen that the chemical polished heat sink with lowest surface roughness and thermal emissivity has the highest temperature, the chemical oxidized heat sink with highest surface roughness and thermal emissivity has the lowest temperature. Temperature of the heat sinks decrease with the surface roughness increase, the biggest difference is about 5 °C with 90 W heating power. In addition, from the fitting curve, it can be seen that the temperature of heat sinks increase almost linearly with the heating power, and the increasing slope of different heat sinks decrease slightly with the roughness increase, so the heat dissipation performance is better with higher surface roughness.

The heat sinks transfer heat to the space by thermal convection and radiation. For thermal convection, it can be calculated by the Newton’s Equation (1):

(1) $$Q_{d}=\alpha_{c}A\Delta t=\alpha_{c}A\left(t_{w}-t_{f}\right)$$

where, *Q_d_* is the convention heat (W), *t_w_* is the surface temperature of object (K), *t_f_* is the temperature of airflow (K), *A* is the surface area of the object involved in heat transfer (m^2^), Δ*t* is the temperature difference between the surface temperature of object and airflow (K), *α_c_* is convective heat transfer coefficient (W/m^2^·K).

For thermal radiation, it can be calculated by the following Equation (2):

(2) $$Q_{r}=\varepsilon \delta A\left(t_{w}^{4}-t_{f}^{4}\right)$$

where, *Q_r_* is the radiation heat (W), *ε* is the thermal emissivity, *δ* is the Stefan–Boltzmann constant, its value is 5.67032 × 10^−8^ W/(m^−2^ × K^−4^).

From these Equations, we can see that the increase of surface area *A* and thermal emissivity *ε* will enhancement the heat dissipation. After surface treatments, the increase of surface roughness also increase the surface area of the heat sinks, and increase the area contact with the air. At the same time, the microstructures on the roughened surfaces will cause local air turbulence, more heat can be taken away by the air. Meanwhile, more heat radiation to the air because of the thermal emissivity increase with the surface roughness increase. With the combined effect of thermal convection and radiation enhancement, thermal equilibrium is established at a lower temperature. The percentage of radiation heat in the total dissipated heat of heat sinks are shown in Figure 6b. It can be seen that the percentage of radiation heat increase with the thermal emissivity and the heating power. The biggest percentage is about 11% with 90 W heating power and chemical oxidation treatment. This is because the radiation heat is related to the difference of the biquadrate of temperature, while convection heat is only related to the difference of temperature. At high temperature, the increase of radiation heat will be greater. Therefore, thermal radiation is very significant at high temperature. Of course, the percentage of radiation heat depends very much on the strength of forced convection. In the natural convection situation, the radiation heat dissipation will account for an even larger percentage. Therefore, thermal radiation cannot be ignored in the heat dissipation design at high temperature, and it is necessary to enhance the thermal radiation by increase surface thermal emissivity. In the future work, the influence of thermal radiation on the overall performance of the heat sinks with good forced convection and natural convection will be studied, and summarize the influence of thermal radiation on the performance of heat sink systematically.

### 3.3. Heat Dissipation Performance of Heat Sinks with Thermal Radiation Coating

Since thermal radiation plays a very important role in heat dissipation, we sprayed a layer of radiant cooling paint with near blackbody emissivity onto the heat sink surfaces, and studied the influence of different surface roughness.

**Figure 7:**
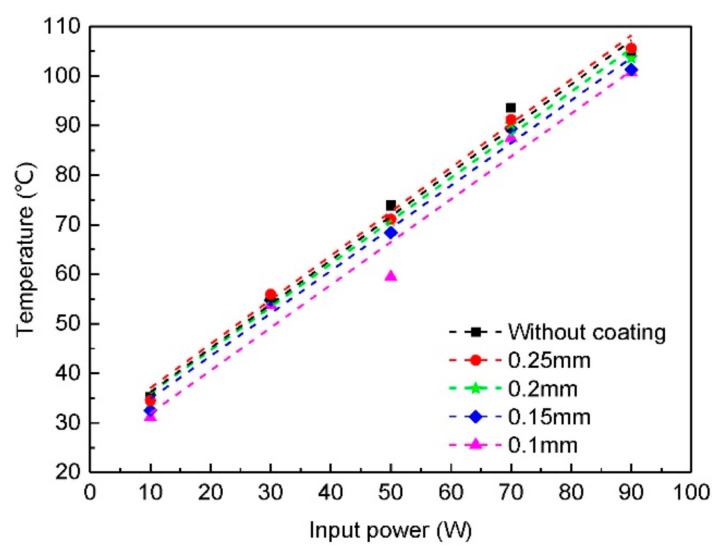
Influence of thermal radiation coating thickness on the temperature of heat sinks.

We first studied the influence of different coating thickness, including 0.1, 0.15, 0.2 and 0.25 mm. The temperatures of untreated heat sinks with different coating thickness in wind tunnel are shown in Figure 7. Form the result, it can be seen that when the coating thickness is 0.1 mm, the temperature of heat sink is about 5 °C lower than the one without coating, so the thermal radiation coating can enhance heat dissipation by increase the thermal radiation. However, when the thickness of the coating increase, the temperature of the heat sinks also increase. When the thickness reached 0.25 mm, the temperature of the heat sink is even higher than the one without coating. At the same time, the temperature increasing slope of heat sinks with different thickness almost has no difference. In this case, the surface roughness of all samples is the same, and the surface thermal emissivity is also the same. However, the total thermal radiation is not the same. The total thermal radiation including three parts, radiation of coating itself, the reflection of external radiation (which is negligible) and the transmitted substrate radiation. When the coating thickness increase, the transmitted substrate radiation will decrease, lead to total thermal radiation reduce. On the other hand, the coating has a relatively high thermal resistance compares to aluminum alloys, when the thickness of the coating increase, the total thermal resistance of the heat sink also increase, the temperature drop from heat sink surface to coating surface is larger, this is bad for both thermal radiation and convention, so the temperature is higher. Therefore, the thickness of the coating should be the minimum thickness which can covers the entire surface.

**Figure 8:**
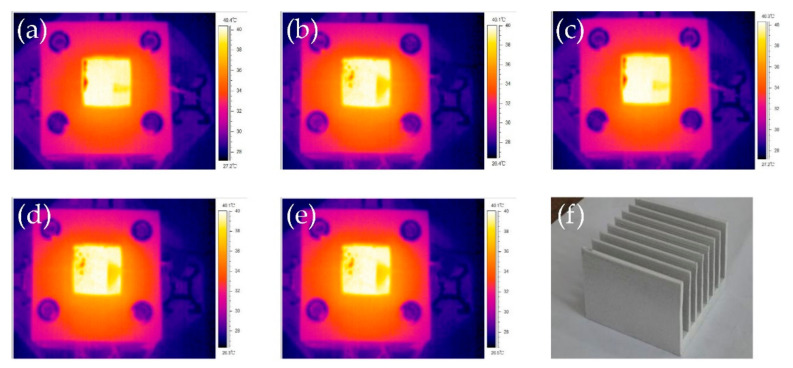
The IR thermal images of heat sink surfaces with thermal radiation coating on different treated surfaces: (**a**) without surface treatment; (**b**) chemical polishing; (**c**) chemical coarsening; (**d**) mechanical shot peening; (**e**) chemical oxidation; (**f**) photo of heat sink with thermal radiation coating.

In order to study the combined effect of the surface microstructure and thermal radiation coating, we coated a 70 μm thick thermal radiation coating onto different treated heat sink surfaces. The IR thermal images of these heat sink surfaces at 40 °C are shown in Figure 8. It can be seen that they all have the same brightness, and the tested thermal emissivity has the same value, which is 0.98. The appearances of the heat sinks with coating are also the same, as shown in Figure 8f. The coating was fabricated by spraying, so it has a certain surface roughness. Since the coating material itself has a very high thermal emissivity, difference in surface roughness will not cause much change.

**Figure 9:**
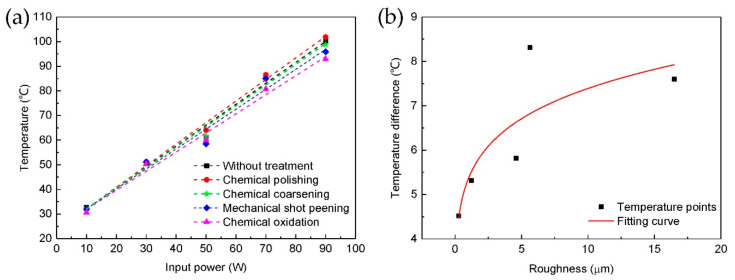
Temperatures of heat sinks with thermal radiation coating in wind tunnel: (**a**) influence of different treated surface under the coating; (**b**) temperature difference of heat sinks with and without coating at different surface roughness (heating power 90 W).

The temperatures of different treated heat sinks with thermal radiation coating in wind tunnel are shown in Figure 9a. At low heating power, the difference between heat sinks is very small. With the heating power increase, the difference also becomes larger. At 90 W, the biggest temperature difference reaches about 10 °C. Therefore, surface microstructure under the thermal radiation coating has influence on the heat dissipation performance of heat sinks. With different surface microstructures, the thermal emissivity does not increase, but the contact surface area between the coating and the treated surface increase significantly. Therefore, heat can conduct from heat sink to coating more efficiently. On the other hand, the coating can partly copy the surface microstructure underneath it, so the coating roughness also increase with the treated surface roughness increase, result in heat convection enhancement. Since all the heat sinks have very high thermal emissivity, the percentage of the radiation heat transfer in the total heat transfer can achieve 36% at highest heating power.

Figure 9b shows the temperature difference of heat sinks with and without coating at different surface roughness, the heating power was 90 W. With the roughness increase, the temperature difference also increase. For heat sinks with low surface roughness, both thermal emissivity and surface roughness increase after coating, but the contact thermal resistance between heat sink surface and coating is high. In this case, the temperature decrease is small. For heat sinks with high surface roughness, both thermal emissivity and surface roughness have less increase after coating, but the contact surface area between the coating and the treated surface increase significantly, so the contact thermal resistance decrease. In this case, the temperature decrease is large. This indicated that the increase of contact surface area plays the main role in the enhancement of heat dissipation performance of heat sinks with coating. Therefore, surface microstructures not only can increase surface thermal emissivity and heat convention, but also can enhance the thermal conductivity between heat sink and thermal radiation coating dramatically.

## 4. Conclusions

In this work, we studied the effect of different treated surfaces on the heat dissipation performance of aluminum alloy heat sinks. Four kinds of treatments, including chemical polishing, chemical coarsening, mechanical shot peening and chemical oxidation, were used to make controllable microstructures on heat sink surfaces. The results show that the thermal emissivity can increase to 2.5 times with surface roughness increase. Benefits from the improvement in thermal emissivity and convection induced by roughened surface, the temperature of the heat sink can reduce by 5 °C under 90 W heating power. Thermal radiation coating also applied onto the heat sink surfaces to improve thermal radiation. We found that the heat sinks with higher surface roughness under the coating have greater improvement because the heat conduction at the coating and heat sink interface was enhanced. In summary, the microstructures can improve the heat dissipation performance of heat sinks significantly by enhancing the thermal convection, radiation and conduction. In the meantime, the proposed microstructures fabricated by the surface treatments on the heat sink surfaces are practicable and easy to implement.

## Figures and Tables

**Table 1:** Surface roughness of heat sink surfaces with different treatments.

Roughness	Without Treatment	Chemical Polishing	Chemical Coarsening	Mechanical Shot Peening	Chemical Oxidation
Ra/μm	1.24 ± 0.05	0.27 ± 0.03	4.6 ± 0.1	5.63 ± 0.07	16.5 ± 0.1
Rz/μm	9.0 ± 0.5	2.3 ± 0.2	27.8 ± 0.6	36.4 ± 0.4	87.8 ± 0.8
Ry/μm	13.8 ± 0.5	3.8 ± 0.3	37.5 ± 1.3	42.1 ± 0.8	107 ± 2

**Table 2:** Thermal emissivity of heat sinks with different surface treatments at 40 °C.

	Without Treatment	Chemical Polishing	Chemical Coarsening	Mechanical Shot Peening	Chemical Oxidation	Thermal Radiation Coating
Emissivity	0.11	0.1	0.16	0.23	0.25	0.98
